# Intraosseous Hemangioma of the Iliac Bone: An Incidental Finding Mimicking Metastatic Disease

**DOI:** 10.7759/cureus.73523

**Published:** 2024-11-12

**Authors:** Mena Louis, Anna Hayden, Nathaniel Grabill, Priscilla Strom

**Affiliations:** 1 General Surgery, Northeast Georgia Medical Center Gainesville, Gainesville, USA

**Keywords:** benign bone tumor, bone biopsy, immunohistochemistry, intraosseous hemangioma, vascular tumor

## Abstract

Intraosseous hemangiomas are rare benign vascular tumors predominantly occurring in the axial skeleton. This case report describes a 44-year-old female with a history of multiple renal stones and benign breast calcifications, who was incidentally found to have a 1.2 cm lucent lesion in the left iliac bone during imaging for nephrolithiasis. Initial concerns for metastatic disease prompted further evaluation, including bone scans and multiple biopsies. The first biopsy revealed benign bone tissue with blood elements, while a subsequent computed tomography (CT)-guided biopsy confirmed the diagnosis of an intraosseous hemangioma through the identification of benign vascular elements and adipose tissue, supported by positive immunohistochemical staining for CD34 and CD31. Accurate diagnosis relies on comprehensive imaging and histopathological evaluation to differentiate them from malignant lesions. Correct identification prevents unnecessary surgical interventions and minimizes associated risks. Conservative management is generally effective for asymptomatic cases, ensuring positive patient outcomes.

## Introduction

Intraosseous hemangiomas are exceptionally rare benign vascular tumors, accounting for less than 1% of all primary bone neoplasms [1]. These lesions predominantly occur in the axial skeleton, with the vertebral bodies and calvarium being the most common sites of origin [2]. The occurrence of intraosseous hemangiomas in the appendicular skeleton, such as the pelvis and long bones, is infrequent, contributing to their overall rarity in clinical practice and making this case particularly noteworthy [2]. This uncommon localization can pose significant diagnostic challenges, as hemangiomas are not typically anticipated in these regions [3].

Clinically, intraosseous hemangiomas are often asymptomatic and are frequently discovered incidentally during imaging studies conducted for unrelated reasons [4]. When symptoms do manifest, they may include localized pain or, in cases involving the spine, neurological deficits due to spinal cord compression [5,6]. Radiologically, these tumors present with diverse imaging characteristics that can vary based on their location and size [7]. On plain radiographs, intraosseous hemangiomas typically appear as well-defined, lytic lesions with a "soap-bubble" or "honeycomb" pattern resulting from internal trabeculations [8]. Advanced imaging modalities such as computed tomography (CT) and magnetic resonance imaging (MRI) provide further delineation of the extent and internal architecture of the lesion [9]. Imaging reveals features such as radiolucent areas with trabecular patterns on CT scans and high signal intensities on both T1- and T2-weighted MRI images, which reflect their vascular nature [7,9].

The differential diagnosis for lytic bone lesions is broad and encompasses both benign and malignant entities, including metastatic carcinoma, multiple myeloma, lymphoma, giant cell tumor, fibrous dysplasia, and osteolytic bone cysts [10]. The nonspecific imaging features of intraosseous hemangiomas often necessitate a comprehensive diagnostic approach to differentiate them from these other conditions [11]. Histopathological examination remains the gold standard for definitive diagnosis, characterized by well-formed vascular channels lined by endothelial cells within a fibrous stroma [12]. Immunohistochemical staining, typically positive for vascular markers such as CD34 and CD31, further aids in distinguishing intraosseous hemangiomas from malignant vascular tumors [13]. Accurate diagnosis is crucial to guide appropriate management strategies, which generally favor conservative observation in asymptomatic cases to avoid unnecessary surgical interventions and associated complications [14].

## Case presentation

A 44-year-old female patient, a former smoker, with a history of multiple renal stones and benign breast calcifications under surveillance presented with symptoms consistent with nephrolithiasis. She had previously undergone subpectoral saline breast implant placement and evaluations for a palpable mass, which were determined to be benign. There was no history of cancer, family history of orthopedic tumors, or other significant comorbidities. Her renal stones were attributed to medication use, and cessation of the medication had prevented further recurrences.

**Figure 1:**
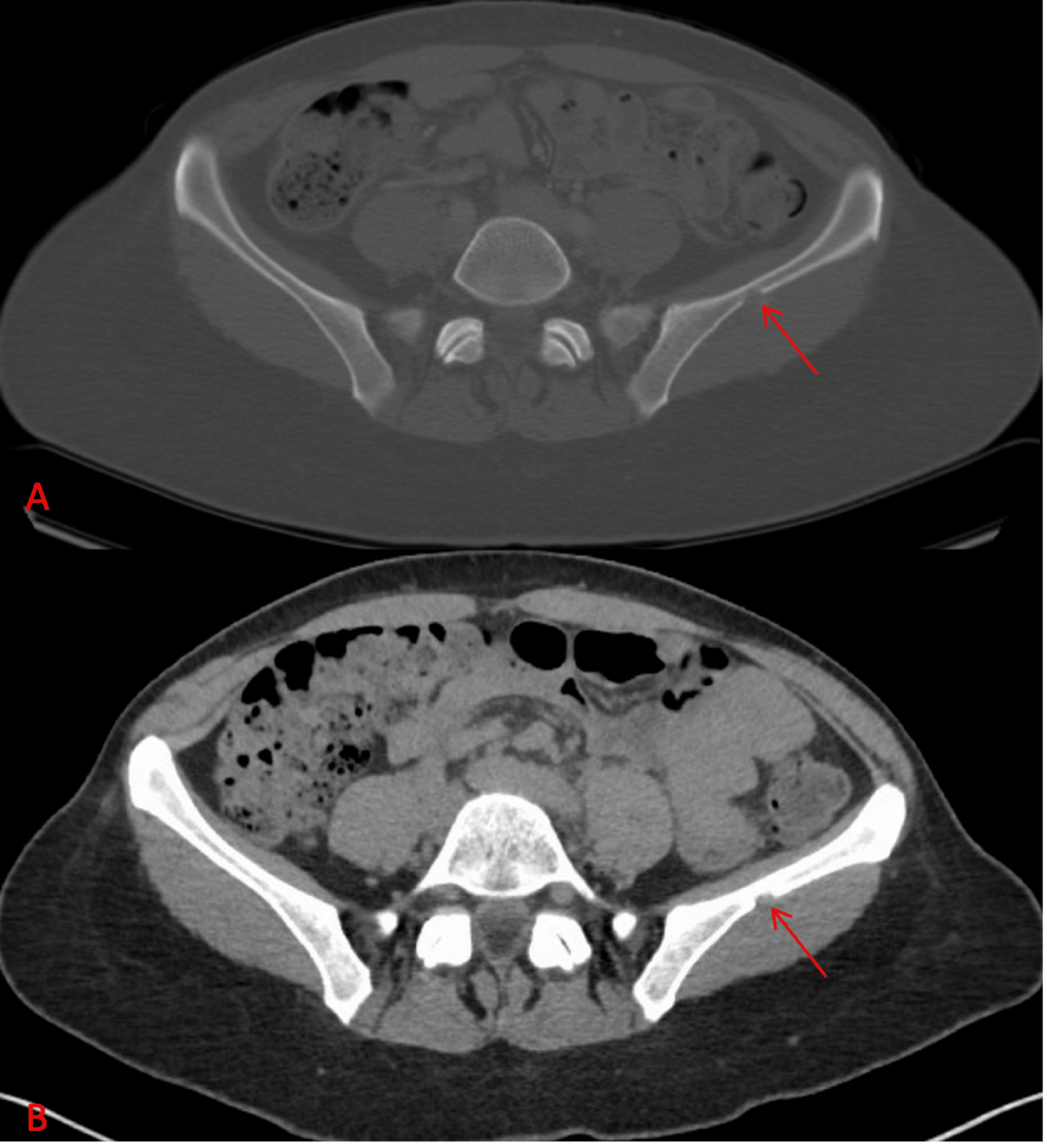
CT of the abdomen and pelvis without contrast (axial bone window (A) and axial abdominal window (B)) with an incidentally found lucent lesion in the left iliac bone (red arrows) CT: computed tomography

**Figure 2:**
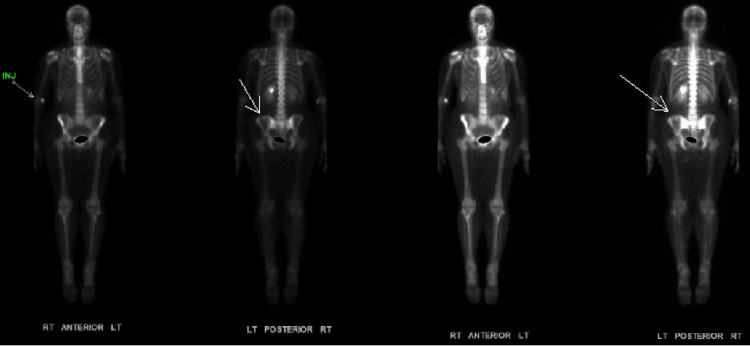
Bone scan with minimal radiotracer uptake in the left iliac wing (white arrows) corresponding to the initial CT image findings with injection site noted in the left antecubital fossa CT: computed tomography

During evaluation for kidney stones, a computed tomography (CT) scan incidentally revealed a 1.2 cm lucent lesion in the left iliac bone (Figure 1). Given her history of breast calcifications, which can be associated with malignancy, the lytic bone lesion raised concerns for potential metastatic disease. Subsequent imaging, including a bone scan and a follow-up CT pelvis, showed features such as posterior cortex breakthrough and an enhancing soft tissue component, which are typically worrisome for metastatic disease. These findings necessitated a thorough and multimodal diagnostic approach to ascertain the nature of the lesion. A bone scan showed minimal uptake in the left iliac wing corresponding to the CT findings (Figure 2). Due to the indeterminate nature of the lesion and the patient's oncologic history, a bone biopsy was recommended.

**Figure 3:**
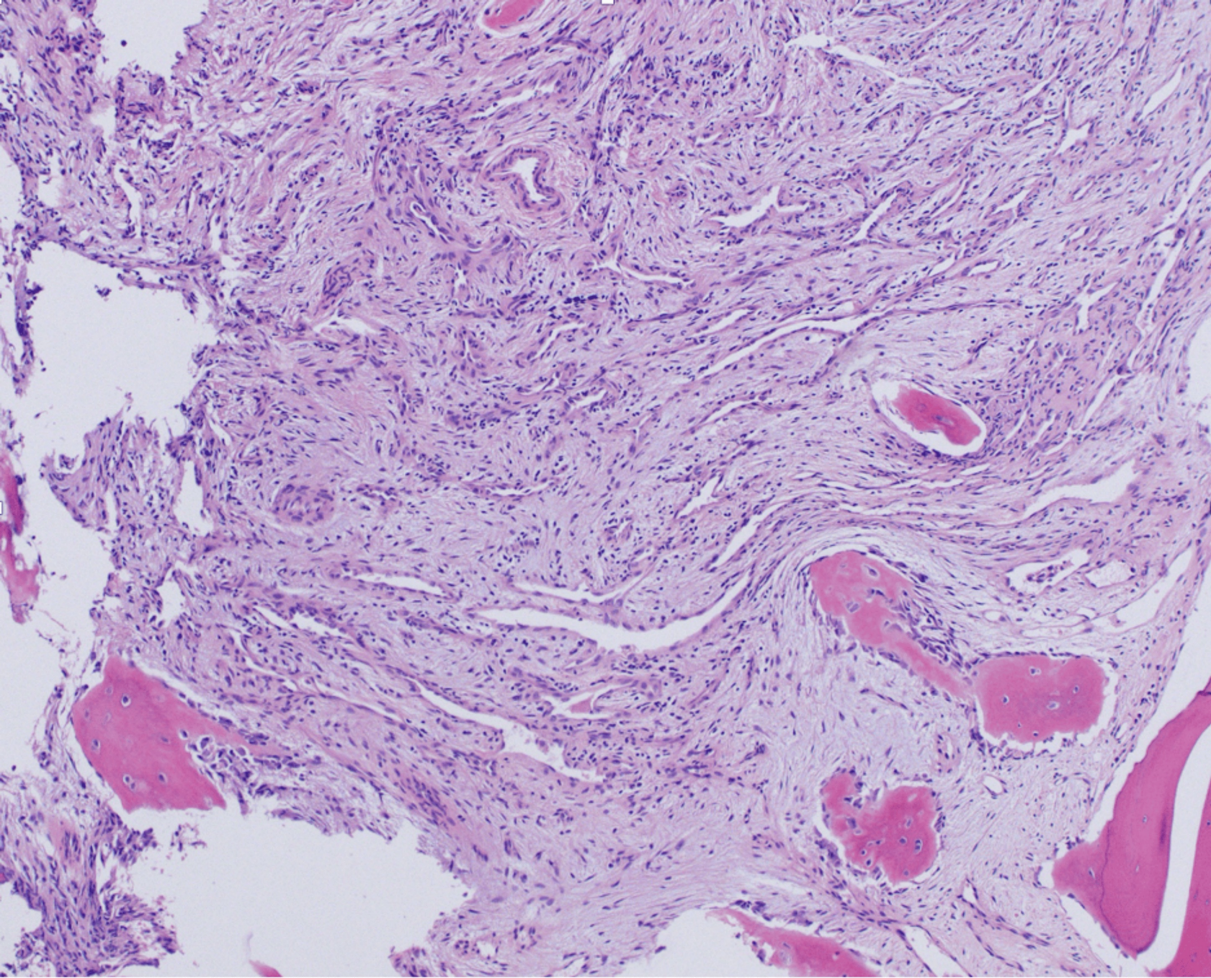
Hematoxylin and eosin-stained histological section of the bone biopsy at 10× magnification displaying cavernous lesions and thin-walled blood vessels characteristic of an intraosseous hemangioma

**Figure 4:**
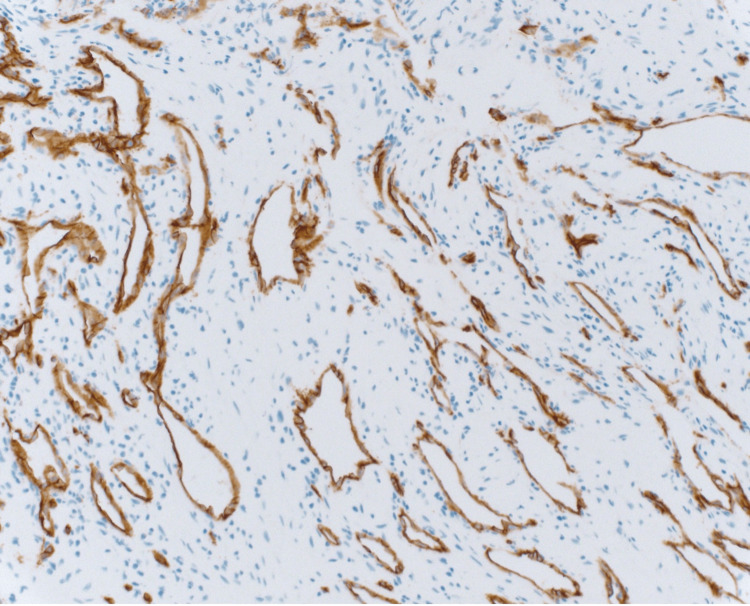
Immunohistochemically stained histological section of the bone biopsy showing positive CD34 expression and confirming a benign vascular tumor

The initial CT-guided bone biopsy of the left iliac wing revealed benign bone tissue with blood elements, with no evidence of malignancy. However, persistent suspicions led to a second CT-guided bone biopsy, which confirmed the diagnosis of an intraosseous hemangioma. The pathology report identified benign vascular structures and adipose tissue, supported by positive immunohistochemical markers CD34 and CD31 (Figure 3 and Figure 4).

Given the asymptomatic nature of the lesion and its low potential for causing structural damage, a conservative management approach was adopted, avoiding unnecessary surgical intervention. The patient was advised to monitor for any new symptoms or changes and to follow up with orthopedics as needed. Throughout the follow-up period, the patient remained asymptomatic regarding the iliac bone lesion, with no reports of pelvic or hip pain or other related complaints. Subsequent imaging confirmed the stability of the hemangioma, validating the conservative management strategy. Additionally, her renal stones were effectively managed with laser lithotripsy and stent placement, and she did not experience any recurrence after discontinuing the causative medication.

## Discussion

The initial presentation of a lucent lesion in the left iliac bone raised significant concerns for metastatic disease, especially given the patient's history of breast calcifications. Lytic bone lesions in the iliac bone are more frequently associated with metastatic carcinoma, multiple myeloma, lymphoma, and other primary bone malignancies [10]. The nonspecific imaging characteristics of intraosseous hemangiomas further complicated the differential diagnosis [7]. On plain radiographs, these lesions may mimic aggressive malignancies with their well-defined, lytic appearance and internal trabeculations [15]. Advanced imaging modalities, including CT and MRI, provide more detailed insights but still may not definitively distinguish hemangiomas from malignant processes without histopathological confirmation [16].

Accurate diagnosis was achieved through a combination of imaging studies and histopathological evaluations. The bone scan demonstrated minimal uptake in the left iliac wing, which, while not definitive, indicated an area of concern consistent with the CT findings. Multiple biopsies were performed to rule out malignancy. The initial bone biopsy revealed benign bone with blood elements, showing no signs of malignancy. The subsequent CT-guided bone biopsy confirmed the presence of an intraosseous hemangioma, characterized by benign vascular elements and adipose tissue. Immunohistochemical staining was negative for GATA-binding protein 3 (GATA-3), pan-keratin, and epithelial membrane antigen (EMA), and positive for CD34 and CD31, supporting the diagnosis of a benign vascular tumor [4]. The utilization of immunohistochemical markers was pivotal in distinguishing hemangioma from malignant vascular tumors, underscoring the importance of comprehensive pathological assessment in such cases [14].

Given the benign diagnosis and the patient's asymptomatic presentation, a conservative management approach was adopted. Orthopedic evaluation determined that the hemangioma was unlikely to cause structural compromise or significant symptoms in the future, warranting no immediate surgical intervention [17]. This decision aligns with current best practices, which advocate for observation and periodic imaging in asymptomatic cases to avoid unnecessary surgical risks [18].

Surgical intervention in intraosseous hemangiomas is generally reserved for symptomatic cases or when there is a risk of structural instability [19]. In highly vascular tumors, surgical resection carries the risk of uncontrollable hemorrhage, making preoperative planning and possible embolization strategies essential to minimize intraoperative risks [20]. In this case, the absence of symptoms and the lesion's stable appearance on follow-up imaging justified the conservative approach.

This case adds to the limited pool of reported intraosseous hemangiomas in the iliac bone, with most documented cases occurring in the spine or skull. Similar cases in atypical locations, such as the zygomatic bone and mandible, have been reported, each presenting unique diagnostic and management challenges based on their anatomical context and symptomatology [16,17]. The successful conservative management in this case reinforces the notion that, when appropriately diagnosed, intraosseous hemangiomas can be managed without invasive procedures, provided they do not pose immediate risks to the patient [17].

Early and accurate identification of intraosseous hemangiomas is crucial to prevent potential surgical complications, particularly hemorrhage due to the tumor's vascular nature [7]. This case exemplifies the value of a meticulous diagnostic workup, including advanced imaging and histopathological analysis, in ensuring patient safety and avoiding unnecessary surgical interventions. By confirming the benign nature of the lesion, the healthcare team was able to adopt a conservative management strategy, thereby minimizing the patient's exposure to surgical risks.

This case report offers significant educational benefits for surgery, family medicine, and emergency medicine residents by highlighting the complexities associated with diagnosing and managing rare bone lesions. For surgery residents, it is important to maintain a broad differential diagnosis when encountering lytic bone lesions and emphasize the necessity of accurate diagnosis to prevent unnecessary surgical interventions [10]. The case provides insight into the utilization of multimodal imaging and biopsy techniques, which are essential skills in preoperative evaluation and surgical planning. Additionally, it highlights the challenges associated with highly vascular tumors, such as the risk of hemorrhage, and the importance of preoperative strategies such as embolization to mitigate these risks [2,11]. The decision-making process between conservative and aggressive management further illustrates evidence-based guidelines that balance the benefits and risks of various treatment approaches, fostering judicious clinical judgment [21]. Moreover, the case demonstrates the value of interdisciplinary collaboration, showcasing how input from orthopedics, radiology, and pathology contributes to optimal patient outcomes.

For family and emergency medicine residents, this case emphasizes the importance of recognizing incidental findings and understanding their potential implications. It provides a framework for managing incidentalomas, including when to initiate further investigation and specialist referral. The ability to interpret radiologic features of bone lesions and distinguish between benign and malignant entities is crucial for effective risk stratification and decision-making in the emergency setting. Furthermore, the case illustrates strategies for effectively communicating complex diagnoses and management plans to patients, which is vital for alleviating anxiety and ensuring adherence to follow-up protocols. By integrating clinical history, imaging, and pathology, emergency medicine residents learn to perform holistic patient assessments, synthesizing information from various sources to formulate comprehensive diagnoses.

## Conclusions

Intraosseous hemangiomas, although rare, should be included in the differential diagnosis of lytic bone lesions to ensure accurate identification. Utilizing advanced imaging techniques and histopathological evaluation is essential for distinguishing these benign vascular tumors from malignant conditions. Accurate diagnosis prevents unnecessary surgical interventions and associated complications, promoting patient safety. Conservative management is often appropriate for asymptomatic cases, highlighting the importance of tailored, evidence-based approaches in clinical practice.
